# Molecular Imprinted Polymer of Methacrylic Acid Functionalised β-Cyclodextrin for Selective Removal of 2,4-Dichlorophenol

**DOI:** 10.3390/ijms15046111

**Published:** 2014-04-10

**Authors:** Hemavathy Surikumaran, Sharifah Mohamad, Norazilawati Muhamad Sarih

**Affiliations:** Department of Chemistry, Faculty of Science, University of Malaya, Kuala Lumpur 50603, Malaysia; E-Mails: hemavathy8605@gmail.com (H.S.); nmsarih@um.edu.my (N.M.S.)

**Keywords:** molecular imprinted polymer, 2,4-dichlorophenol, β-cyclodextrin, removal, kinetics, isotherm

## Abstract

This work describes methacrylic acid functionalized β-cyclodextrin (MAA-βCD) as a novel functional monomer in the preparation of molecular imprinted polymer (MIP MAA-βCD) for the selective removal of 2,4-dichlorophenol (2,4-DCP). The polymer was characterized using Fourier Transform Infrared (FTIR) spectroscopy, Brunauer-Emmett-Teller (BET) and Field Emission Scanning Electron Microscopy (FESEM) techniques. The influence of parameters such as solution pH, contact time, temperature and initial concentrations towards removal of 2,4-DCP using MIP MAA-βCD have been evaluated. The imprinted material shows fast kinetics and the optimum pH for removal of 2,4-DCP is pH 7. Compared with the corresponding non-imprinted polymer (NIP MAA-βCD), the MIP MAA-βCD exhibited higher adsorption capacity and outstanding selectivity towards 2,4-DCP. Freundlich isotherm best fitted the adsorption equilibrium data of MIP MAA-βCD and the kinetics followed a pseudo-second-order model. The calculated thermodynamic parameters showed that adsorption of 2,4-DCP was spontaneous and exothermic under the examined conditions.

## 1. Introduction

Water quality control is one of the most critical issues in environmental analytical chemistry today. Phenols are one of the consequential toxic pollutants classified under organic aromatic contaminants [1]. Phenols can be released into the environment directly and indirectly [2], thus they appear to be one of the problematic groups globally. Chlorophenols are hazardous wastes mainly produced during chemical processing of pesticides, herbicides, paint, paper production and wood preservations [3]. The chlorination of natural waters for disinfection produce small amounts of chlorinated phenols [4]; particularly, 2,4-dichlorophenol (2,4-DCP)s are very common and have been highlighted as priority pollutants to be monitored in the aquatic environment by the US EPA in the Clean Water Act [5–7]. Carcinogenic and mutagenic effects to living things may occur because 2,4-DCP can incite perturbances in the cellular structure of phospholipid bilayers [8]. Generally, 2,4-DCPs are weak acids, which are resistant to biodegradation. Before discharging 2,4-DCP into receiving water bodies, they have to be decomposed so that the deleterious effects of bio magnifications to aquatic flora and fauna can be obviated [9]. Complicated problems such as unpleasant odor and taste in drinking water, inhibition of the mundane activities of microbial population in wastewater treatment plants etc., also occur upon existence of 2,4-DCP in bodies of water. Prolonged consumption of excessive phenol concentrations will results in loss of appetite, malnutrition, headache, rapid tiredness and severe insomnia in human beings [10]. Concentration of chlorophenols as low as 0.1 mg·L^−1^ can exhibit harmful effects in drinking water [11].

Several physical, chemical and biological methods including activated carbon adsorption [12], ion exchange [13,14], biodegradation [15,16], advanced oxidation processes such as photo-Fenton reactions and ozonation [17–20] have been reported for removal of chlorophenols. Among these methods, adsorption is a well-known removal technique for organic compounds from water [21] due to its simple design, facile operation and relatively low cost [22]. The utilization of activated carbon as adsorbents for the removal of phenolic compounds [23] is a very common practice. However, adsorptions using activated carbon have high treatment costs, including possibilities of causing secondary pollution and considerable loss during chemical or thermal regeneration [24]. Moreover, these methods are normally non-selective and this has led researchers to continue searching for more reliable, cost-effective and highly selective materials for removal of chlorophenols. In this context, molecular imprinting techniques could serve as a versatile platform for creating polymer matrix with molecule-specific recognition properties besides its simple, inexpensive and stable nature [25].

Molecularly imprinted polymers (MIPs) are new kinds of smart materials with promising selective molecular recognition abilities. The functional monomer and cross-linker are copolymerized in the existence of a template molecule. Extraction of the template molecule from the prepared polymer brings out binding sites that are similar to the target molecule in size, shape and physicochemical properties, which can differentiate the template from its structural analogues [26,27]. MIPs have been attractive in many fields such as sensors [28], analytical applications [29,30], biomedical applications and enantiomeric separations. Till now, MIPs were mostly synthesized utilizing typical functional monomers, methacrylic acid (MAA) and non-polar solvents such as porogen, in which interactions such as hydrogen bonds and electrostatic interaction were only responsible for imprinting effect. It is hard to use these MIPs directly in aqueous samples, because interactions between the template and monomers will be disturbed by water molecules [31]. Therefore, a novel water insoluble imprinted material was prepared via polymerization of new functional monomer, methacrylic acid functionalized β-cyclodextrin (MAA-βCD) and trimethylolpropane trimethacrylate (TRIM) as a cross linker in the presence of 2,4-DCP (template molecule) to overcome this problem.

Cyclodextrins (CDs) are torus-shaped cyclic oligosaccharides consisting of several α-1,4-linked d-glucopyranose units. β-cyclodextrin (β-CD) has been chosen for this study since its hydrophobic cavity can accommodate a variety of compounds to form inclusion complexes by various kinds of intermolecular interactions [32]. In recent years, they have received considerable attention in molecular imprinting techniques [33,34]. This inclusion interaction within β-CD cavities is one of the driving forces to be considered in the case of β-CD MIPs besides interaction with the cross-linking network [35]. To the best of our knowledge, only limited numbers of studies had described the use of 2,4-DCP as template for MIP synthesis in literature [36,37]. Pan and co-workers recently reported on MIP microspheres using β-cyclodextrin/attapulgite composites for adsorption of 2,4-DCP from aqueous solution. But the idea of the present study is totally different from their work since modified β-CD was utilized as a functional monomer rather than using composites as support in imprinting technique. In the current work, the primary objectives are: (1) to synthesize and characterize new functional monomers (MAA-βCD) and imprinted polymers (MIP MAA-βCD); (2) to evaluate the influences of adsorption parameters (*i.e.*, pH, contact time, initial concentrations of 2,4-DCP and temperature); (3) to establish a kinetic and isotherm model that best described the adsorption process of 2,4-DCP and (4) to calculate the thermodynamic parameters of the adsorption of 2,4-DCP by MIP MAA-βCD.

## 2. Results and Discussion

### 2.1. Characterization of MIP MAA-βCD and NIP MAA-βCD

#### 2.1.1. Fourier Transform Infrared (FTIR) Analysis

**Figure 1.**
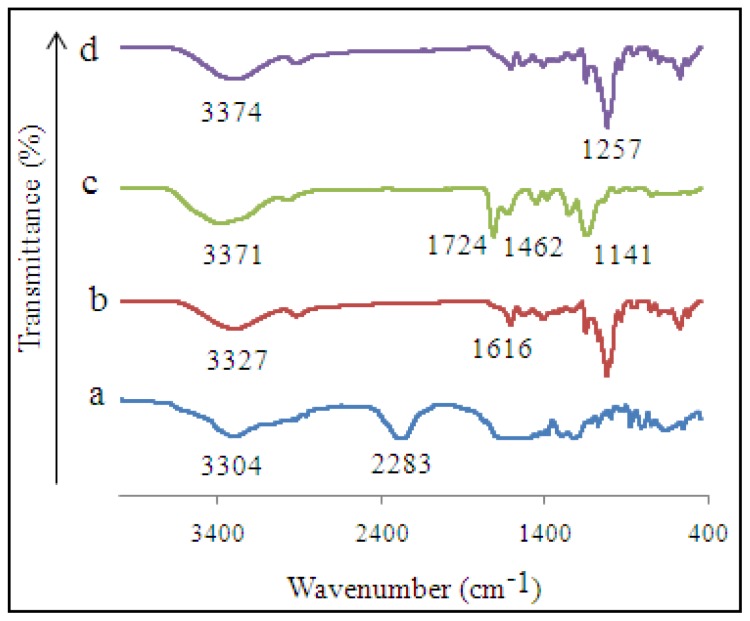
IR spectra of (a) MAA-TDI; (b) MAA-βCD; (c) MIP MAA-βCD; and (d) NIP MAA-βCD.

FTIR analysis permits identification of the functional groups on the adsorbent surface in the range 400–4000 cm^−1^ [38]. The IR spectra of MIP MAA-βCD, NIP MAA-βCD and their monomers are shown in Figure 1. The spectrum of MAA-βCD (Figure 1b) shows the complete disappearance of N=C=O, (isocyanate peak of toluene-2,4-diisocyanate,TDI) at 2283 cm^−1^ and formation of a new carbamate group (–NH) at 3327 cm^−1^ after reacting with β-CD. The absence of N=C=O group shows that it has been reacted with –OH group of β-CD. Presence of a C=C bond of MAA at 1616 cm^−1^ indicate that the double bond of MAA is intact. This is very important because this double bond will be used in the cross-linking process with TRIM during polymerization. Therefore, this result is evidence for the formation of new monomer (MAA-βCD) formed between MAA-TDI and β-CD. Compared with the spectrum of NIP MAA-βCD (Figure 1d), there were only slight changes such as broadening of some bands and small spectral shifts in the spectrum of MIP MAA-βCD. Strong peaks around 3371, 2953, 1724, 1462, 1257 and 1141 cm^−1^ were observed in the spectrum of MIP MAA-βCD (Figure 1c). The peak at 3371 cm^−1^ was due to the formation of –NH group between –OH (β-CD) and N=C=O (MAA-βCD) from monomer. The band at around 2953 cm^−1^ was due to the symmetric and asymmetric C–H stretching vibrations. The bands around 1462 cm^−1^ were suggested to be the aromatic ring stretching vibrations of 2,4-DCP. The band located at 1257 cm^−1^ assigned to the stretching vibration of the C–OH of alcoholic groups and carboxylic acids from MAA-βCD. A band centered at 1141 cm^−1^ was due to C–O stretching of 2,4-DCP. The obvious absorption band (C=O) of TRIM located at 1724 cm^−1^ indicates the cross-linking reaction successfully occurred. However, the new absorption bands were not observed in the spectrum of MIP MAA-βCD when 2,4-DCP was loaded onto the polymer. Only small shifts were observed in some absorption bands. This is an indication for inclusion complex formation between 2,4-DCP and β-CD in MIP MAA-βCD during the imprinting process.

#### 2.1.2. Particle Size and Brunauer-Emmett-Teller (BET) Analysis

MIP and NIP particles were subjected to small angle x-ray scattering to determine the particle size. The size of the MIP particle after extraction is 122.6 A while for the NIP particle it is 311.8 A. Particle size of MIP after extraction was smaller than NIP. This suggests that after extraction MIP MAA-βCD had more vacancy sites and porosity surface for adsorption of 2,4-DCP whereas NIP MAA-βCD did not contain them.

**Figure 2.**
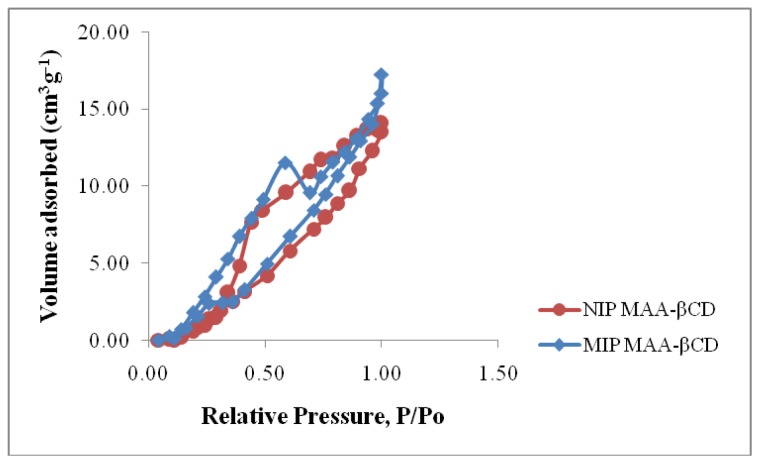
Nitrogen adsorption/desorption profiles of MIP MAA-βCD and NIP MAA-βCD.

Meanwhile, nitrogen adsorption/desorption analysis was used to evaluate Brunauer-Emmett-Teller (BET) surface area, total pore volume and pore size. The total pore volume and average pore diameter of the polymers was quantified using Barrett-Joyner-Halenda (BJH) method. The corresponding isotherm profiles are presented in Figure 2; the shape of their N_2_ adsorption/desorption isotherm was a mixture of types I and IV, suggesting that both MIP MAA-βCD and NIP MAA-βCD had a mixed microporous and mesoporous structure. The BET surface area, total pore volume and average pore size of MIP MAA-βCD were 2.441 m^2^·g^−1^, 0.022 cm^3^·g^−1^, 1.587 nm respectively, while these parameters were not obviously different from those of NIP MAA-βCD (2.225 m^2^·g^−1^, 0.019 cm^3^·g^−1^, 1.465 nm). Therefore, the distinguishable adsorption properties of MIP MAA-βCD and NIP MAA-βCD were not caused by the morphological differences, but due to the imprinting effect of template molecule. Referring to the IUPAC definition, MIP MAA-βCD and NIP MAA-βCD mainly possessed micropores (pore size < 2 nm) [38]. MIP MAA-βCD had many imprinted cavities and should have more surface area and pore volume. However, the functional monomers (MAA-βCD) were distributed in an orderly fashion at the surface of pores in the MIP MAA-βCD via the imprinting process. As a result, the MIP MAA-βCD could absorb much more water molecules and had less surface area and pore volume.

#### 2.1.3. Morphological Structure

**Figure 3.**
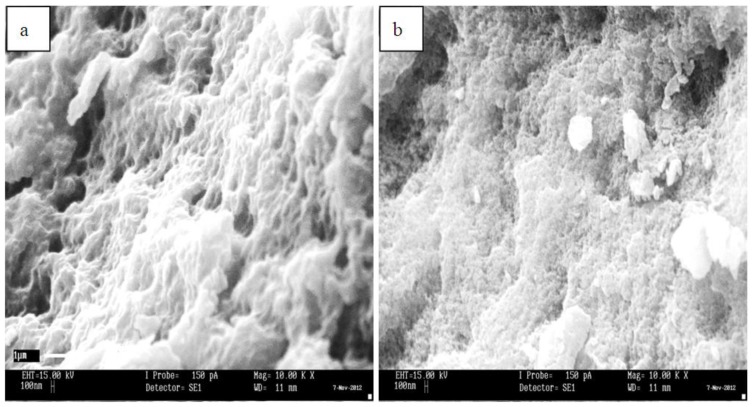
FESEM images of (**a**) MIP MAA-βCD and (**b**) NIP MAA-βCD. (Magnification: ×10,000).

Field Emission Scanning Electron Microscopy (FESEM) was used to characterize MIP MAA-βCD and NIP MAA-βCD morphologically as shown in Figure 3. Significant differences can be noticed in the FESEM images of the polymers. The surface of the MIP MAA-βCD exhibits a more porous structure than NIP MAA-βCD. The regular structure of the NIP MAA-βCD was due to the fact that no specific binding sites had been created for the polymer. The cavities in the MIP MAA-βCD were probably caused by the structure of the 2,4-DCP molecules. This phenomenon explains well the experimental results of the MIP MAA-βCD, which gave high adsorption capacity to 2,4-DCP.

### 2.2. Effect of pH on the Adsorption of 2,4-DCP

**Figure 4.**
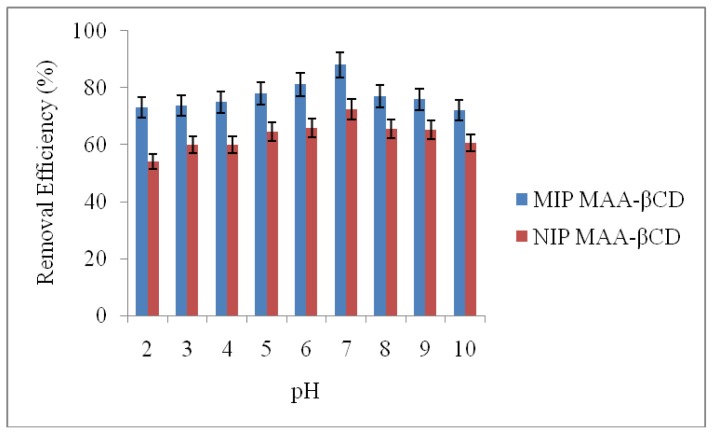
Effect of pH on the adsorption of 2,4-DCP onto MIP MAA-βCD and NIP MAA-βCD (Adsorption conditions: adsorbent dose = 20 mg, initial concentration = 10 mg·L^−1^, solution volume = 10 mL, T =298 K, time = 2 h).

The effect of pH on the adsorption of 2,4-DCP onto MIP MAA-βCD and NIP MAA-βCD was examined (Figure 4). The result showed that the adsorption was not pH-dependent. The initial pH of the solutions was adjusted from pH 2 to 10. Removal of 2,4-DCP by MIP MAA-βCD was not significantly different than that of NIP MAA-βCD at all pH solutions studied. The removal efficiency of 2,4-DCP increased gradually with the increase in initial pH and reached optimum value of 83% at pH 7 and decreased slightly at basic pH region (8–10). Therefore all the other sorption tests in this work were performed at pH 7.

At neutral pH, the adsorbed 2,4-DCP was mostly in the non-ionized form. The sorption mechanism was probably dominated by van der Waals force, inclusion complex formation and hydrogen bonding simultaneously. Some researchers had proved that 2,4-DCP could form inclusion complex with β-CD [39,40]. Since MIP MAA-βCD does not have functional groups with proton dissociation, the adsorption of 2,4-DCP is thus pH-independent and mainly due to hydrophobic interactions.

### 2.3. Effect of Initial 2,4-DCP Concentrations on the Adsorption Process

**Figure 5.**
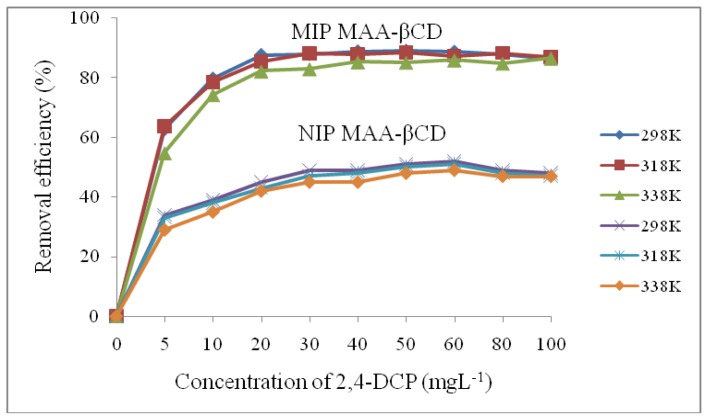
Effect of initial concentrations on the adsorption of 2,4-DCP onto MIP MAA-βCD and NIP MAA-βCD under different temperatures (Adsorption conditions: adsorbent dose = 20 mg, solution volume = 10 mL, time = 45 min, pH = 7.0).

Figure 5 shows the plot of removal percentage *versus* initial concentrations for the adsorption of 2,4-DCP onto MIP MAA-βCD and NIP MAA-βCD at three different temperatures (298, 318 and 338 K). The isotherm data of 2,4-DCP showed that the binding isotherm at first elevates sharply, indicating that few numbers of readily accessible sites are available in the beginning for adsorption. However, after achieving equilibration, a plateau is reached demonstrating that no more sites are available for further adsorption as the adsorbent was saturated. The increase in initial concentration from 5 to 100 mg·L^−1^ leads to an increase in removal efficiency generally from 62% to about 87%. The effect of temperature on the percentage of removal is not very obvious. The percentage of removal decreases slightly with the increase in temperature. This implies that the adsorption of MIP MAA-βCD towards 2,4-DCP is an exothermic process [35].

### 2.4. Adsorption Isotherms

The adsorption isotherm is studied to describe the interactive behavior between the adsorbate and the adsorbent. Isotherm data analysis is important for estimating the adsorption capacity and for describing the adsorbent surface properties and affinity. Four most commonly used isotherm models, Langmuir [41], Freundlich [42], Temkin [43] and Dubinin-Radushkevich [44] were used to describe relationships between the amount of 2,4-DCP adsorbed onto MIP MAA-βCD and its equilibrium concentration in solutions at different temperatures. The applicability of the isotherm models was studied by judging the correlation coefficients, *R*^2^ values. The Freundlich isotherm model is most frequently employed to describe sorption onto heterogeneous surfaces from aqueous solution and reversible adsorption, which is not restricted to monolayer formations. It can be expressed in linear form as Equation (1):

(1) $$\text{log qe}=\text{log }{\text{K}}_{\text{F}}+(1/\text{n})\;\text{log Ce}$$

where *K*_F_ (mg·g^−1^) (L·mg^−1^)^1/^*^n^* and 1/*n* are Freundlich constants related to adsorption capacity and adsorption intensity of the adsorbent respectively. The value of n indicates a favorable adsorption when 1 < *n* < 10 and it is more favorable as 1/*n* < 1 [45].

The Langmuir isotherm model is predicated on the postulation that a structure of the adsorbent is homogeneous, where all sorption sites are similar and energetically equivalent. The general Langmuir equation is given as Equation (2):

(2) $$\frac{\text{Ce}}{\text{qe}}=\frac{1}{{\text{Q}}_{\text{m}}\text{b}}+\frac{1}{{\text{Q}}_{\text{m}}}\text{Ce}$$

where *C*e is the equilibrium concentration of the 2,4-DCP (mg·L^−1^), q*e* is the equilibrium adsorption capacity (mg·g^−1^), *b* is the Langmuir adsorption constant (L·mg^−1^) and Qm is the maximum adsorption amount (mg·g^−1^), which can be obtained from the slope and intercept of a plot *C*e/q*e versus C*e. The favorability of an adsorption process is determined in terms of Langmuir isotherm, “*R*_L_” a dimensionless constant referred as separation factor or equilibrium parameter [46] is calculated using Equation (3):

(3) $${\text{R}}_{\text{L}}=\frac{1}{1+{\text{bC}}_{0}}$$

where *b* is the Langmuir isotherm constant (L·mg^−1^) and C_0_ is the highest initial 2,4-DCP concentration (mg·L^−1^). The *R*_L_ value indicates the favorability of adsorption system. When 0 < *R*_L_ < 1.0, it shows a good adsorption.

Temkin and Pyzhev explain the effects of indirect adsorbent/adsorbate interactions on adsorption isotherms. This isotherm postulates that (1) the adsorption heat of all the molecules in the layer reduces with coverage due to adsorbent-adsorbate interactions and that (2) the adsorption is explained by an even distribution of binding energies, up to some maximum binding energy [43]. The linear form of the Temkin isotherm model is described in Equation (4):

(4) qe=(RT/b) ln (A)+(RT/b) ln Ce

where *B* = RT/*b*, *A* = *K*_T_ is the equilibrium binding constant (L·mg^−1^) related to the maximum binding energy and constant *B* is corresponds to the heat of adsorption. The values of *K*_T_ and *B* are determined using the intercept and slope of the linear plot of *q*e *versus* ln *C*e.

Another model is Dubinin-Radushkevich (D-R) isotherm model, which is shown in the following linearized form as Equation (5):

(5) $$\text{ln Qe}=\text{ln }{\text{Q}}_{\text{m}}-{\text{K}}_{\text{DR}}{ɛ}^{2}$$

The work required to remove a molecule to infinity from its location in the sorption space, which is not subject to control of temperature, is referred to as the Polanyi sorption potential, ɛ. The Polanyi sorption theory assumes fixed volume of sorption sites proximate to the sorbent surface and presence of sorption potential over these sites [47]. Polanyi sorption potential, ɛ is equal to Equation (6):

(6) ɛ=RT ln (1+1/Ce)

*R* is a gas constant (8.314 J mol·K^−1^) and T is the temperature in Kelvin. This model explains about the sorption energies within this space, which is heterogenous. The constant *K*_DR_ is related with free energy (*E*, kJ·mol^−1^) of adsorption per molecule of the adsorbate when it is transferred to the surface of the solid from infinity in the solution and the value of sorption energy, *E*, can be correlated to *K*_DR_ utilizing the following relationship as shown in Equation (7). A plot of ln *q*e *versus* ɛ^2^ enables the constants, *K*_DR_ and *E* to be determined.

(7) $$\text{E}={\left(2{\text{K}}_{\text{DR}}\right)}^{-1/2}$$

Linearized forms of Langmuir, Freundlich, Temkin and Dubinin-Radushkevich isotherm models were applied to fit the equilibrium data. The adsorption isotherm constants for MIP MAA-βCD at three temperatures were listed in Table 1. The experimental data of 2,4-DCP adsorption onto MIP MAA-βCD fitted well to the Freundlich equation, which was statistically confirmed by greater *R*^2^ values more proximate to unity (0.999). *K*_F_, Freundlich constant for adsorption capacity showed a decrease tendency with the rise of temperature determined as 2.244, 0.475 and 0.404 for 298, 318 and 338 K, respectively. Meanwhile the n values (indicator for adsorption intensity) calculated from the Freundlich model were in the range of 1 < *n* < 10, indicating favorable adsorption. The adsorption of 2,4-DCP is most favorable at 298 K followed by 318 K since the values of 1/*n* calculated less than 1 as shown in Table 1. This result suggests that the 2,4-DCP could be readily absorbed by MIP MAA-βCD. Temkin and Dubinin-Radushkevich isotherm models also showed relatively good agreement with the *R*^2^ > 0.94 and *R*^2^ > 0.97 respectively. The calculated value of *K*_DR_ < 1.0 represents the rough surface with many cavities. This finding further supports the fit of isotherm data for the Freundlich model, which suggests a heterogeneous surface.

While the Langmuir model is used for a description of homogeneous systems, the adsorption interaction on the monolayer coverage of 2,4-DCP onto MIP MAA-βCD particles showed very poor fit with *R*^2^ of only 0.512. Therefore, the Freundlich model was the most appropriate model to describe the adsorption of 2,4-DCP onto MIP MAA-βCD. This suggests that the adsorption occurred on a heterogeneous surface, which also directly indicates that the surface of MIP MAA-βCD is heterogeneous. The heterogeneity is due to the various functional groups on the MIP MAA-βCD as well as the multiple adsorbent-adsorbate interactions such as hydrogen bonding and formation of inclusion complexes between β-CD and 2,4-DCP. Moreover, it has been reported in the scientific literature that adsorption equilibriums of phenol and chlorophenols fitted Freundlich isotherm better than the Langmuir [24]. As a conclusion, the adsorption equilibrium data fitted the isotherm models in the order of: Freundlich > Dubinin-Radushkevich > Temkin > Langmuir.

### 2.5. Adsorption Kinetics

#### 2.5.1. Adsorption Kinetic Models

The rate at which adsorbates are removed from aqueous solution onto adsorbent surface was described by adsorption kinetics. The adsorption kinetics of 2,4-DCP was evaluated using three kinetic models, namely the pseudo-first-order, pseudo-second-order and Elovich models. The pseudo-first-order model is widely used and this kinetic equation postulates that the rate of change of solute uptake with time is directly proportional to the difference in saturation concentration and the amount of solid adsorbed with time [48].

(8) $$\frac{\text{dqt}}{\text{dt}}={\text{k}}_{1}\;(\text{qe}-\text{qt})$$

When q*t* = 0 at *t* = 0, Equation (8) can be integrated into the following equation Equation (9):

(9) $$\text{log }(\text{qe}-\text{qt})=\text{log qe}-\frac{{\text{k}}_{1}}{2.303}\text{t}$$

where q*e* and q*t* are the amount of 2,4-DCP adsorbed (mg·g^−1^) at equilibrium and at time *t*, *t* is the contact time (min) and *k*_1_ is the rate constant (min^−1^). The values of *k*_1_ and q*e* were calculated from a plot of log (q*e* − q*t*) *versus t*. A pseudo-second-order kinetic equation was defined by Ho and McKay [49] as:

(10) $$\frac{\text{dqt}}{\text{dt}}={\text{k}}^{2}{\left(\text{qe}-\text{qt}\right)}^{2}$$

Integrating Equation (10) and noting that when q*t* = 0 at *t* = 0, the linearized form of the pseudo-second-order model given as Equation (11):

(11) $$\frac{\text{t}}{\text{qt}}=\frac{1}{{\text{k}}^{2}{\text{q}}^{2}\text{e}}+\frac{1}{\text{qe}}\text{t }\;\;\text{where},\text{h}={\text{k}}^{2}{\text{q}}^{2}\text{e}$$

where *h* represents the initial adsorption rate (mg·g^−1^ min), and *k*_2_ is the pseudo-second-order rate constant (g·mg^−1^ min). The values of q*e*, *k*_2_ and *h* can be obtained by a linear plot of *t*/q*t versus t*.

The elovich model represented in Equation (12) as follows:

(12) $$\text{qt}=\frac{1}{\beta }\text{Ln}(\alpha \beta )+\frac{1}{\beta }\text{Ln t}$$

where α (mg·g^−1^·min^−1^): initial sorption rate and β (g·mg^−1^): related to the extended of surface coverage and activation energy for chemisorption. This model is applicable when plot of q*t versus* ln *t* represents a straight line [50].

#### 2.5.2. Validation of Kinetic Models

The suitability of the model to describe the adsorption kinetics was further justified predicated on the normalized standard deviation value, Δ*q* (%) and relative error (%) which is defined as Equations (13) and (14):

(13) $$\Delta q\;(\%)=\sqrt{\frac{{\left[(q_{\text{exp}}-q_{\text{cal}})/q_{\text{exp}}\right]}^{2}}{N-1}}\times 100$$

(14) $$\text{Relative error }(\%)=\frac{\left|\text{qe},\text{cal}-\text{qe},\text{exp}\right|}{\text{qe},\text{exp}}\times 100$$

where *N* is the number of data points, q*e*_exp_ and q*e*_cal_ (mg·g^−1^) are the experimental and calculated adsorption capacity respectively. Generally, the model fits better if the value of Δ*q* is lower [51].

#### 2.5.3. Intraparticle Diffusion

For a solid-liquid adsorption process, the solute transfer is usually characterized through external diffusion, intraparticle diffusion, or both [52]. Basically, the adsorption kinetics is controlled by three steps related with the adsorption of solute from the solution by an adsorbent. These include (1) film diffusion; (2) intraparticle or pore diffusion; and (3) sorption into interior sites. The last step is very fast and considered to be negligible, hence the overall rate of adsorption is controlled by the slowest step, which would be either film or pore diffusion [53]. The kinetic data can be used to confirm the presence of intraparticle diffusion to determine whether intraparticle diffusion is the sole rate-inhibiting step. Weber and Morris described a functional relationship of intraparticle diffusion [54] in Equation (15)

(15) $$\text{qt}={\text{k}}_{\text{i}}{\text{t}}^{1/2}+\text{C}$$

where q*t* (mg·g^−1^) is the uptake of 2,4-DCP at time *t* (min), *k*_i_ (mg·g^−1^·min^1/2^) is the intraparticle diffusion rate constant and the intercept *C*, obtained by extrapolation of the linear plot of q*t versus t*^1/2^ is a constant that gives a conception of the thickness of the boundary layer.

#### 2.5.4. External Diffusion

External diffusion or film diffusion model described as follows in Equation (16):

(16) $$\text{Ln}\frac{\text{Ct}}{{\text{C}}_{0}}=-{\text{k}}_{\text{ext}}\text{t}$$

where *C*_0_ and *C*_t_ (mg·L^−1^) are the concentrations of the solute in the initial solution and in the liquid phase at time *t*, respectively, and *k*_ext_ (min^−1^) is a diffusion rate parameter. The plot of ln (*C*_t_/*C*_0_) against *t* should give a linear line with zero intercept if external diffusion is applicable [50].

Adsorption kinetics explains the rate of adsorbate uptake on MIP MAA-βCD and controls the equilibrium time. Pseudo-first-order, pseudo-second-order and Elovich kinetic models were applied to gain insight about the kinetics of adsorption process of 2,4-DCP. Intraparticle and external diffusion models were utilized to study the diffusion mechanism of the adsorption. The kinetic parameters calculated from the slopes and intercepts of the plots of these models were listed in Table 2. If the plots of the log (q*e* − q*t*) *versus t* were to be linear with good *R*^2^, the pseudo-first-order equation is appropriate to the experimental data of the adsorption process. On the other hand, if the correlation coefficient for the pseudo-second-order model higher and its calculated equilibrium adsorption capacity (q*e* calculated) is consistent with q*e* experimental, these suggests that the pseudo-second-order adsorption mechanism is predominant and that the overall rate of the adsorption process is controlled by the chemisorption process [49]. It can be clearly seen that the pseudo-second-order model best fitted with extremely high *R*^2^ (0.999) almost equal to unity and a good agreement is also achieved between the q*e* calculated and q*e* experimental values which was 4.587 and 4.585 respectively as shown in Table 2, further supports the statement mentioned earlier which is the adsorption process follows chemisorption when calculated equilibrium adsorption capacity (q*e* calculated) is consistent with experimental value (q*e* experimental). The pseudo-first-order kinetic curve did not give a good fit to the experimental kinetic data with relatively lower *R*^2^ value (0.670) and the experimental q*e* value (4.585) did not agree with the calculated ones (0.561). So, the adsorption of 2,4-DCP onto MIP MAA- βCD obviously follows second-order reaction.

The best fit to the pseudo-second-order indicated that the adsorption mechanism might depend on both the adsorbate and the adsorbent and the rate-limiting step is chemisorption involving valence forces through the sharing or exchange of electrons [38]. The solute molecules can react with two kinds of adsorption sites. First, 2,4-DCP can be imprinted by active sites in the polymer network. Second, 2,4-DCP can form inclusion complex with β-CD in molecular recognition process. The kinetic data was additionally well described by the Elovich model. The plot of q*t versus* ln *t* yields the *R*^2^ value of 0.886 for this model. The α and β value is 1.799 g·mg^−1^ and 25.498 mg·g^−1^·min^−1^ respectively and these values were obtained from the slope and intercept of the plot. The models fits the kinetic data on the order of pseudo-second-order kinetics > elovich > pseudo-first-order kinetics. The accuracy and validity of the reported kinetic models were compared using *R*^2^ values, normalized standard deviation Δ*q* (%) and relative error (%) which were listed in Table 2. It was found that the pseudo-second-order model fits the kinetic adsorption curve better than other models since the calculated Δ*q* (%) and relative error (%) for this model were extremely low in comparison.

External and intraparticle diffusion models were used to gain an insight into the mechanism of the adsorption process and to predict the rate-controlling step. The Weber and Morris model is adopted since this is a widely used intraparticle diffusion model. The values of *k*_i_ and *C* obtained are 0.117 and 3.288 respectively. The *R*^2^ of 0.525 was obtained for this model. In order to say that the intraparticle diffusion is the only rate-controlling step, the plot of q*t versus t*^1/2^ should be linear and pass through the origin. However in this study, the plot did not pass through the origin and this is probably due to the difference in the beginning and final stages of adsorption in terms of the mass transfer rate [54]. The external diffusion model was studied to investigate whether other mechanisms were involved. A linear plot with the *R*^2^ (0.459) and the intercept value of −1.136 suggested that the external diffusion is also not the only rate-limiting step in the adsorption process. Hence, it can be concluded that both external and intraparticle diffusion occurred simultaneously during the adsorption of 2,4-DCP onto MIP MAA-βCD. Rate constants for both diffusion models were given in Table 2.

**Figure 6.**
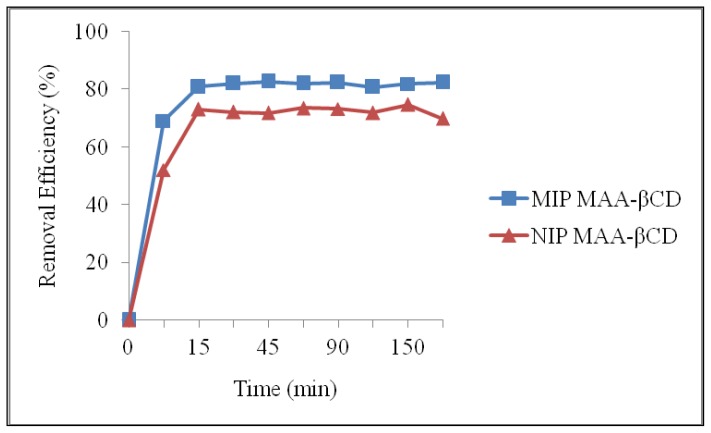
Effect of time on the adsorption of 2,4-DCP onto MIP MAA-βCD and NIP MAA-βCD (Adsorption conditions: adsorbent dose = 20 mg, initial concentration = 10 mg·L^−1^, solution volume = 10 mL, *T* = 298 K, pH = 7.0).

Figure 6 depicts the effect of contact time (0–180 min) of MIP MAA-βCD and NIP MAA-βCD towards the removal of 2,4-DCP. Compared with NIP MAA-βCD, it was obvious that a much higher adsorption capacity was achieved on MIP MAA-βCD. The adsorption process of 2,4-DCP could be divided into two steps. In the beginning step, the adsorption rate was fast; rapid increases in removal percentage was achieved during the first 45 min and reached equilibrium with the maximum percentage removal of 83%. The fast adsorption at the initial stage may be due to the availability of the uncovered surface area and the remaining active sites on the MIP MAA-βCD [55]. Hence, 2,4-DCP molecules can reach the imprint sites easily and quickly during the rebinding step. Therefore, MIP MAA-βCD showed good site accessibility for 2,4-DCP and equilibrium was achieved quickly. After 45 min, there is no significant difference in adsorption. This is attributed to the fact that at this stage the imprinted sites for 2,4-DCP molecules maybe fully saturated. As a conclusion, higher adsorption efficiency was obtained in a shorter time for 2,4-DCP removal.

### 2.6. Adsorption Thermodynamics

Adsorption thermodynamics were studied to gain idea about the adsorption behaviors [56]. Thermodynamic behavior of the adsorption of 2,4-DCP on MIP MAA-βCD was evaluated by the thermodynamic parameters such as change in Gibb’s free energy (Δ*G*°), enthalpy (Δ*H*°) and entropy (Δ*S*°). The Gibbs free energy change Δ*G*°, denoting the degree of the spontaneity of the adsorption process is calculated using Equation (17):

(17) $$\Delta G^{\circ }=\Delta H^{\circ }-T\Delta S^{\circ }$$

The enthalpy change (Δ*H*°) and the entropy change (Δ*S*°) are calculated using Equation (18) respectively:

(18) $$\text{ln K}=\frac{\Delta {\text{S}}^{\circ }}{\text{R}}-\frac{\Delta {\text{H}}^{\circ }}{\text{RT}}$$

where *K* (^−1^) is from Langmuir equation, *R* is the gas constant (8.314 ) and *T* is the temperature in Kelvin [57]. The values of ln *K* were plotted against 1/*T*, the Δ*H*° and Δ*S*° values were calculated from the slope and intercept of the plot.

The dependence of adsorption with temperature has been evaluated and the numerical values of Δ*H*° and Δ*S*° were obtained from the slope of plot ln *K versus* 1/*T*. The calculated thermodynamic parameters were listed in Table 3. The calculated Gibbs free energy change, Δ*G*° values are −3.501, −3.734, −3.966 kJ·mol^−1^ at 298, 318 and 338 K respectively. The Δ*G*° value generally increases as temperature elevates. This is due to the increase in feasibility of adsorption at higher temperatures. Negative Δ*G*° values for studied temperatures, revealed the thermodynamically spontaneous and feasible nature of this adsorption. The calculated enthalpy change, Δ*H*° is −32.090 kJ·mol^−1^. The negative value of Δ*H*° demonstrating the adsorption of MIP MAA-βCD towards 2,4-DCP is an exothermic process which agrees with the equilibrium isotherm data. Δ*H*° value also gives information on the type of adsorption, which can be either physical or chemical [54]. The obtained ΔH° is not in the range of physisorption. Therefore, the Δ*H*° value shows that the adsorption of 2,4-DCP onto MIP MAA-βCD was taken place via chemisorption. The calculated entropy change, Δ*S*° value is 11.640 J·mol^−1^·K^−1^. Generally positive Δ*S*° value suggests the adsorbate at the solid/solution interface organized more randomly. It is additionally supposed that the change of Δ*S*° value is related to the displacement of the adsorbed water molecules by the adsorbate [56]. Hence in this study, the positive Δ*S*° value suggested incrementation in randomness of adsorbate-solution interface during the adsorption process and displacement of water molecules by 2,4-DCP on MIP MAA-βCD.

### 2.7. Selective Adsorption of MIP MAA-βCD towards 2,4-DCP

Competitive adsorptions of 2,4-DCP, 4-CMP, 2-CP, 4-NP and 2-NP from their mixtures were studied using MIP MAA-βCD and NIP MAA-βCD to test the selectivity and the details of the experiment were given in Section 3.6. Selectivity experiments over the interference compounds can be evaluated by using the following equation:

(19) $$K_{d}=\frac{[Ci-Cf]\times \text{v}}{M}$$

where *K*_d_ is referred as distribution coefficient, *Ci* and *Cf* is the initial and final concentration. *v* (L) is the volume used and *M* (g) is the weight of MIP. The selectivity coefficient for the binding of 2,4-DCP in the presence of interfering species can be estimated by using the following equation:

(20) $$k=\frac{K_{d}(2,4-\text{DCP})}{K_{d}(\text{phenolic})}$$

where *k* is the selectivity coefficient and (phenolic) represents the 4-CMP, 2-CP, 4-NP and 2-NP compounds. Effect of imprinting on selectivity is estimated by comparing *k* values of the imprinted polymers with other phenolic compounds. *k*′, a relative selectivity coefficient can be defined as:

(21) $$k^{\prime }=\frac{k_{\text{imprinted}}}{k_{\text{non}-\text{imprinted}}}$$

MIP MAA-βCD had higher selectivity to its template (2,4-DCP) compared to NIP MAA-βCD. Table 4 summarizes *K_d_*, *k*, *k*′ values of 4-CMP, 2-CP, 4-NP and 2-NP with reference to 2,4-DCP. The NIP MAA-βCD is not as selective as MIP MAA-βCD probably due to the non-specific interaction and absence of imprinted sites. The results of the present study suggested that MIP MAA-βCD can selectively remove 2,4-DCP even in the presence of interferences. So, it can be concluded that the selectivity of MIP MAA-βCD was due to the existence of imprinted cavities with definite size, shape and distinct binding interactions with template molecules [58].

### 2.8. Comparison with Other Adsorbents

Table 5 summarizes the comparison of the adsorption capacities of different types of adsorbents for 2,4-DCP that has been reported in literature. The high adsorption capacity, q*e* of MIP MAA-βCD for 2,4-DCP in this study is relatively comparable with some previous works. Thus, MIP MAA-βCD can be considered as one of the promising adsorbents for removing 2,4-DCP from aqueous solutions.

### 2.9. Possible Interaction between 2,4-DCP and MAA-βCD

**Figure 7.**
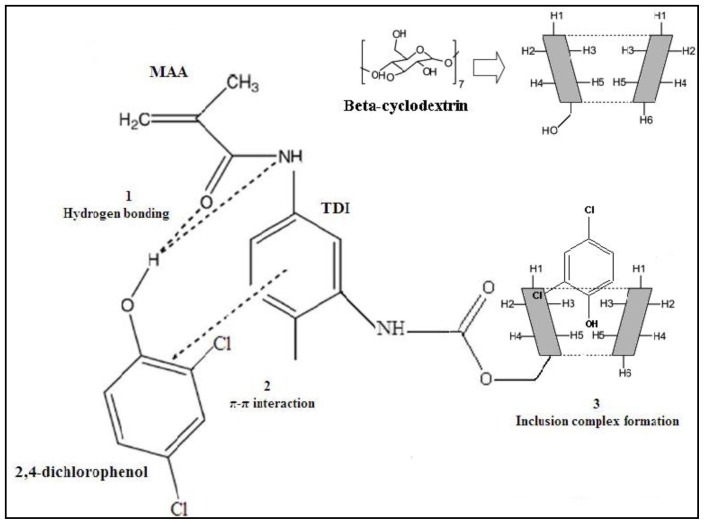
The proposed interaction mechanism between functional monomer, MAA-βCD and 2,4-DCP (template).

There are various types of possible interactions between MAA-βCD monomer and 2,4-DCP as shown in Figure 7. (1) Formation of hydrogen bonding between hydrogen atom of 2,4-DCP and nitrogen or oxygen atom in MAA-βCD monomer; (2) π-π interaction between the aromatic ring of 2,4-DCP and the benzene ring of TDI in MAA-βCD monomer; (3) Inclusion complex formation between β-CD cavity in MAA-βCD and 2,4-DCP molecule. Hydrophobic effects play an important role in the process of adsorption. Due to the hydrophobic nature of the 2,4-DCP and the hydrophobic core of β-CD cavity, the 2,4-DCP molecule could be inserted into the cavity of the β-CD residues in the rebinding process in aqueous media. ^1^H NMR analysis of MAA-βCD monomer, 2,4-DCP and inclusion complex (MAA-βCD-DCP) was carried out (Refer to Figure S1). A comparison of ^1^H NMR chemical shifts for 2,4-DCP, MAA-βCD and their inclusion complex was shown (Refer to Table S1). ^1^H NMR result reveals that obvious chemical shifts occurred at H3 and H5 protons, which are located inside the β-CD cavity, and that the OH proton of 2,4-DCP had penetrated into the cavity. Even though similar inclusion complex formation can occur in NIP MAA-βCD, the absence of imprinted sites for 2,4-DCP and different orientation of the adsorption sites such as β-CD cavity from MIP MAA-βCD explains the decrease in adsorption efficiency of NIP MAA-βCD. In conclusion, the presence of triple interactions between MAA-βCD and 2,4-DCP is responsible for high recognition ability of the MIP MAA-βCD, which resulted from modification of MAA monomer using β-CD.

## 3. Experimental Section

### 3.1. Chemicals

Methacrylic acid (MAA), toluene-2,4-diisocyanate (TDI), benzoyl peroxide (BPO) and trimethylolpropane trimethacrylate (TRIM) were obtained from Sigma Aldrich, USA. β-cyclodextrin (β-CD) is purchased from MP Biomedical, France. 2,4-dichlorophenol (2,4-DCP), 2-chlorophenol (2-CP), 4-chloro-3-methylphenol (4-CMP), 4-nitrophenol (4-NP) and 2-nitrophenol (2-NP) were supplied by Fluka, Switzerland. All the chemicals were of the highest quality available and used without further purification. Dimethylacetamide (DMAC) solvent used was of analytical reagent grade. Ultrapure water (18 MΩ cm, Millipore Corporation, Rockland, MA, USA) was used for the preparation of aqueous solutions. Stock solutions were prepared by dissolving 1.0 g of 2,4-DCP in 1.0 L of methanol and was stored at 4 °C in the dark to prevent photodegradation. Working solutions were prepared daily by diluting the stock solutions using water just before use.

### 3.2. Instruments

Infrared spectra (IR) were recorded at 4000–400 cm^−1^ by using a Fourier Transform Infrared (FT-IR) spectrometer (Perkin-Elmer RX1, Perkin-Elmer Waltham, MA, USA). The Brunauer-Emmett-Teller (BET) surface area and porous properties of MIP MAA-βCD and NIP MAA-βCD were determined from the nitrogen adsorption-desorption analysis at 77 K on surface area analyzer (Quantachrome, Boynton Beach, FL, USA). The samples were previously degassed at 393 K, overnight. BET equation was used to calculate the specific surface area. Field Emission Scanning Electron Microscopy (FESEM) images were performed on Quanta FEG 450 (FEI, Hillsboro, OR, USA) to analyze the morphology and surface structure of the polymers. The particle size analysis was done using small angle X-ray scattering with a PANalytical EMPYREAN (Almelo, The Netherlands) X-ray diffractometer (voltage, 40 kV; current 100 mA). UV-Vis spectrophotometer (Shimadzu, Japan) equipped with 1 cm quartz cells was used for the quantification of the 2,4-DCP at λ_max_ = 285 nm. For selectivity studies, mixture of phenols was analyzed using an Agilent 7890A Gas Chromatography (GC) system with an Agilent 5975C Series GC/FID from Agilent Technologies Inc. (Santa Clara, CA, USA). The GC column used was a Agilent HP-5MS column (30 m × 0.32 mm i.d. and 0.25 μm film thickness). The GC oven temperature program used was as follows: 35 °C held for 1 min, 35 °C/min to 220 °C, held for 8 °C ·min^−1^. The injector temperature was 250 °C (splitless). The detector temperature was 300 °C. The carrier gas was Helium with a flow rate of 1.1 mL·min^−1^ and nitrogen was used as the make-up gas with a flow rate of 32.4 mL·min^−1^. The data were processed using Agilent Chemstation (Agilent Technologies, Santa Clara, CA, USA).

### 3.3. Synthesis of MAA-βCD Monomer

**Figure 8.**
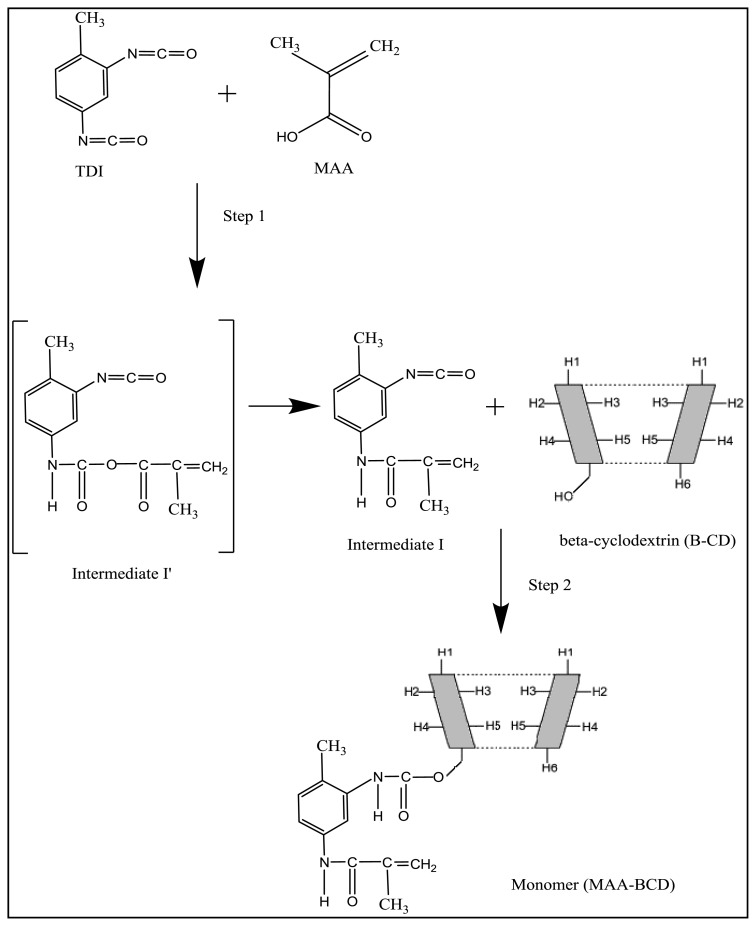
The synthetic route of the MAA-βCD monomer.

The molar concentration is chosen in such a way to react only one –OH group of β-CD. The stoichiometry ratio is 0.5 M MAA: 1 M TDI: 0.5 M β-CD. The MAA-βCD monomer was synthesized according to some literature with slight modification [66–68]. Firstly, MAA and toluene-2,4-diisocyanate (TDI) were allowed to mix in 40 mL DMAC; 0.1% dibutyltin dilaurate (catalyst) was then added and the solution was stirred magnetically for 1 h at room temperature in the presence of nitrogen gas. A few drops of this solution were subjected to FTIR analysis. Lastly, a calculated amount of β-CD was added to this solution together with 10 mL DMAC and further stirred for about 2 h. The final product was also characterized using FTIR. The possible mechanism for this reaction is shown in Figure 8.

From Figure 8, it can be seen that intermediate I’ contains an anhydride and a carbamate group had formed at the consumption of carboxyl group in MAA. Intermediate I’ is unstable and it converts to methacrylic amide containing an isocyanate, N=C=O group as shown in Intermediate I. At first, only one of the N=C=O group in TDI took part in the reaction. The remaining unreacted N=C=O in Intermediate I used for further isocyanation with one of the primary –OH of β-CD to yield MAA-βCD (monomer). This monomer later on will be utilized in imprinting technique to produce MIP MAA-βCD and NIP MAA-βCD. Structural analysis confirmed that the new monomer was successfully prepared using this two-step isocyanation.

### 3.4. Synthesis of MIP MAA-βCD and NIP MAA-βCD

**Figure 9.**
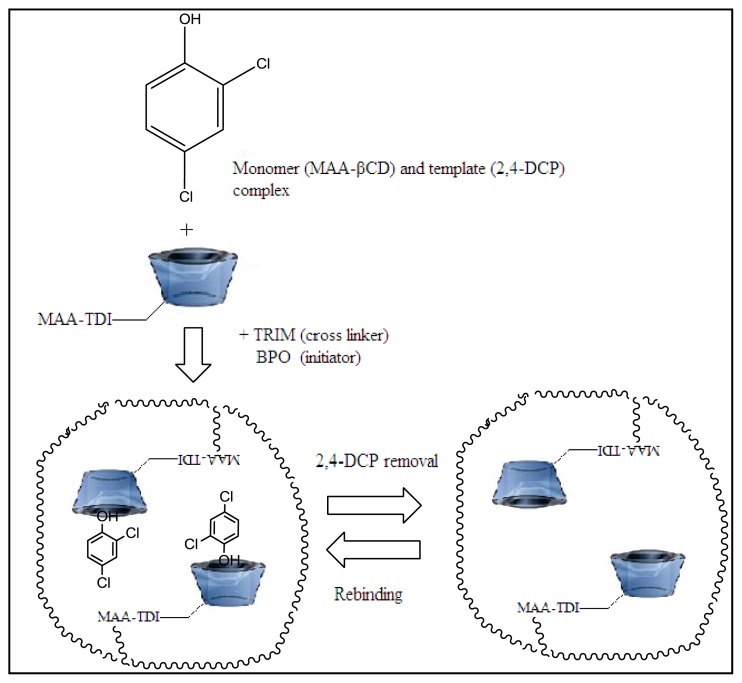
Molecular imprinting of MIP MAA-βCD using 2,4-DCP as template.

Imprinted and non-imprinted polymers were prepared according to the procedure reported earlier [69]. Molecular imprinted polymer (MIP MAA-βCD) was prepared using the non-covalent approach. The template, 2,4-DCP (0.14 mmol) was dissolved in 60 mL DMAC in a flask. The functional monomer, MAA-βCD (0.56 mmol), the cross-linker, TRIM (2.80 mmol) and the initiator benzoyl peroxide (BPO, 1 g) were then added to the flask. The flask was sealed after purging with nitrogen for 10 min. The contents were allowed to polymerize in a water bath at 70 °C for 24 h. The obtained bulk polymer was crushed and ground to get regular sized particles. The template molecules were removed using methanol/acetic acid (3:1 *v*/*v*) solution six times. The particles were then washed a few times with water until no more 2,4-DCP was detected. The evidence for the complete removal of the template molecule is given in the Figure S2. Non-imprinted polymer (NIP MAA-βCD) was prepared in a same way as MIP MAA-βCD, but without the addition of template molecule. The molecular imprinting technique using 2,4-DCP is shown in Figure 9.

### 3.5. Batch Adsorption Studies

The effect of solution pH, contact time, concentration and temperature on the removal of 2,4-DCP was investigated. Adsorption experiments were performed by shaking 20 mg of MIP MAA-βCD with 10 mL of 10 mg·L^−1^ 2,4-DCP solution at 298 K in a water bath shaker (Daihan Scientific, Seoul, Korea). Continuous mixing was provided during the experiment, with a constant agitation speed of 180 rpm. The solution pH was measured by pH meter (Hanna Instruments, Carrolton, TX, USA) and the initial pH of the solutions were adjusted from pH 2 to 10 by adding 0.1 M HCl or 0.1 M NaOH solutions as needed. Kinetic studies were used to study the effect of contact time and to calculate the kinetic parameters. To determine the effect of time on the removal of 2,4-DCP, the adsorption process was conducted for the time interval of (5, 15, 30, 45, 60, 90, 120, 150 and 180) min. Meanwhile to study the equilibrium isotherm of the adsorption, various initial concentrations of 2,4-DCP were studied (5, 10, 20, 30, 40, 50, 60, 80, 100 mg·L^−1^) under three different temperatures (298, 318, 338 K). For this purpose, the pH values of the solutions studied were adjusted to pH 7 and were shaken in a water bath for 45 min. Finally at the end of contact time, all the sample solutions were filtered and the residual 2,4-DCP concentration in each supernatant solutions was analyzed using a UV-Vis spectrophotometer at a maximum wavelength of 285 nm. Each test was done three times as parallel experiments and the experimental data was the mean of their results. The 2,4-DCP uptake at equilibrium defined as adsorption capacity, q*e* (mg·g^−1^) and the percentage of removal, % removal were calculated by using Equations (22) and (23) respectively:

(22) $$\text{Qe}=\frac{\text{V}\times ({\text{C}}_{0}-\text{Ce})}{\text{M}}$$

(23) $$\%\;\text{Removal}=\frac{\left({\text{C}}_{0}-\text{Ce}\right)}{{\text{C}}_{0}}\times 100$$

where *C*_0_ and *C*e are the initial and equilibrium concentrations of 2,4-DCP (mg·L^−1^). *V* is the volume of the solution (*L*) and *M* is the weight of MIP MAA-βCD (g).

### 3.6. Selectivity Experiments

**Figure 10.**
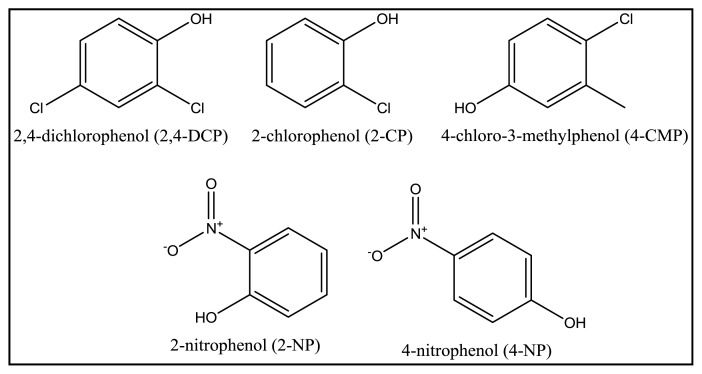
2,4-dichlorophenol and its structural analogues.

To evaluate the selectivity of MIP MAA-βCD for 2,4-DCP, selective adsorption experiments were carried out in aqueous solution containing mixtures of phenols (2,4-DCP, 2-CP, 4-CMP, 2-NP and 4-NP). Their molecular structures are shown in Figure 10. A 10 mL solution containing 10 mg·L^−1^ (of each phenol) was mixed together and treated with MIP MAA-βCD (20 mg) at 298 K. The concentration of the phenol compounds after treatment was measured by GC-FID.

## 4. Conclusions

In this work, the molecular imprinting technique has been successfully utilized in conjunction with bulk polymerization method to produce MIP MAA-βCD. This novel material showed high affinity, selectivity and fast kinetics for the adsorption of 2,4-DCP. The adsorption of 2,4-DCP onto MIP MAA-βCD is pseudo-second-order kinetics, which indicates a chemisorption process. The equilibrium data was best fitted to the Freundlich model with a correlation coefficient value of (*R*^2^ > 0.99) and the order of isotherm fit is as follows: Freundlich > Dubinin-Radushkevich > Temkin > Langmuir. MIP MAA-βCD could be considered a promising sorbent for the removal of chlorophenols from aqueous solutions and could provide possibilities for applications in water treatments, especially for the removal of trace phenols from drinking water. Future studies will be made to verify whether the observed behavior is generally applicable.

## Supplementary Information



## Figures and Tables

**Table 1.** Isotherm constants of four isotherm models for 2,4-DCP adsorption onto MIP MAA-βCD.

Isotherm Constants	Temperature (K)		

	298	318	338
Langmuir			
Q max (mg·g^−1^)	−90.909	−125	−90.909
*b* (L·mg^−1^)	−0.004	−0.003	−0.004
*R*_L_	1.65	1.42	1.59
*R*^2^	0.541	0.557	0.512

Freundlich			
*K*_F_ (mg·g^−1^(L·mg^−1^)^1/^*^n^*)	2.244	0.475	0.404
*n*	1.025	1.009	0.975
1/*n*	0.976	0.991	1.026
*R*^2^	0.999	0.995	0.999

Temkin			
*K*_T_ (L·mg^−1^)	0.071	0.071	0.071
*B*	20.18	19.6	18.54
*R*^2^	0.962	0.948	0.967

Dubinin-Radushkevich			
*K*_DR_ (mol^2^ kJ^2^)	0.023	0.024	0.021
*E* (kJ·mol^−1^)	4.662	4.564	4.879
*R*^2^	0.981	0.992	0.978

**Table 2.** Kinetic parameters for 2,4-DCP adsorption onto MIP MAA-βCD.

Kinetic Model	Constants	MIP MAA-βCD
Pseudo-first-order model	q*e*, experimental (mg·g^−1^)	4.585
	q*e*, calculated (mg·g^−1^)	0.561
	*k*_1_ (min^−1^)	0.016
	Δ*q* (%)	43.882
	Relative error (%)	87.764
	*R*^2^	0.67

Pseudo-second-order model	q*e*, experimental (mg·g^−1^)	4.585
	q*e*, calculated (mg·g^−1^)	4.587
	*h* (mg·g^−1^ min )	4.367
	*k*_2_ (g·mg^−1^ min)	0.207
	Δ*q* (%)	0.017
	Relative error (%)	0.046
	*R*^2^	0.999

Elovich model	q*e*, experimental (mg·g^−1^)	4.585
	q*e*, calculated (mg·g^−1^)	4.243
	B (g·mg^−1^)	1.799
	α (mg·g^−1^·min^−1^)	25.498
	Δ*q* (%)	3.725
	Relative error (%)	7.451
	*R*_2_	0.886

Intraparticle diffusion	q*e*, experimental (mg·g^−1^)	4.585
	*C* (mg·g^−1^)	3.288
	*k*_i_ (mg·g^−1^·min^1/2^)	0.117
	Δ*q* (%)	12.651
	Relative error (%)	28.288
	*R*^2^	0.525

External Diffusion	*k*_ext_ (min^−1^)	0.003
	*C*	−1.136
	*R*^2^	0.459

**Table 3.** Thermodynamic adsorption parameters.

Sorbent	Thermodynamic Parameters			

	Δ*H*° (kJ·mol^−1^)	Δ*S*° (J·mol^−1^·K^−1^)	*T* (K)	Δ*G*° (kJ·mol^−1^)
MIP MAA-βCD	−32.09	11.64	298	−3.501
			318	−3.734
			338	−3.966

**Table 4.** Selectivity parameters of the MIP MAA-βCD and NIP MAA-βCD.

Phenols	MIP MAA-βCD		NIP MAA-βCD		*k*′
	
	*K_d_* (mg·g^−1^)	*k*	*K_d_* (mg·g^−1^)	*k*	
2,4-DCP	5.19		2.76		
4-CMP	2.54	2.04	2.34	1.18	1.73
2-CP	1.77	2.93	1.94	1.42	2.06
2-NP	1.9	2.73	1.79	1.54	1.77
4-NP	2.65	1.96	2.25	1.23	1.59

**Table 5.** Comparison of the adsorption capacities for 2,4-DCP by MIP MAA-βCD in this study and various adsorbents reported in literature.

Adsorbent	Adsorption Capacity, q*e* (mg·g^−1^)	Reference
MIP MAA-βCD	45.67	This study
Maize cob carbon	17.94	[59]
Palm pith carbon	19.16	[60]
Oil palm empty fruit bunch carbon	27.25	[61]
Paper mill sludge	4.49	[62]
Aged refuse in biofilter	1.53	[63]
Poly HEMA microbead	16.1	[64]
Blast furnace sludge	29.1	[65]
β-CD Attalpugite composites	19.04	[36]
β-CD epichlorohydrin polymer	15.7	[39]
