# Supplementary material for: Molecular Imprinted Polymer of Methacrylic Acid Functionalised β-Cyclodextrin for Selective Removal of 2,4-Dichlorophenol
Source: Int J Mol Sci. 2014 Apr 10;15(4):6111–36. doi: 10.3390/ijms15046111 (PMC4013619; doi:10.3390/ijms15046111)
Supplement: Supplementary file 1 [file ijms-15-06111-s001.pdf]

Supplementary Information

Figure S1. ^{1}H NMR spectrum of (**a**) Inclusion complex (MAA-βCD-DCP); (**b**) 2,4-DCP and (**c**) MAA-βCD monomer.

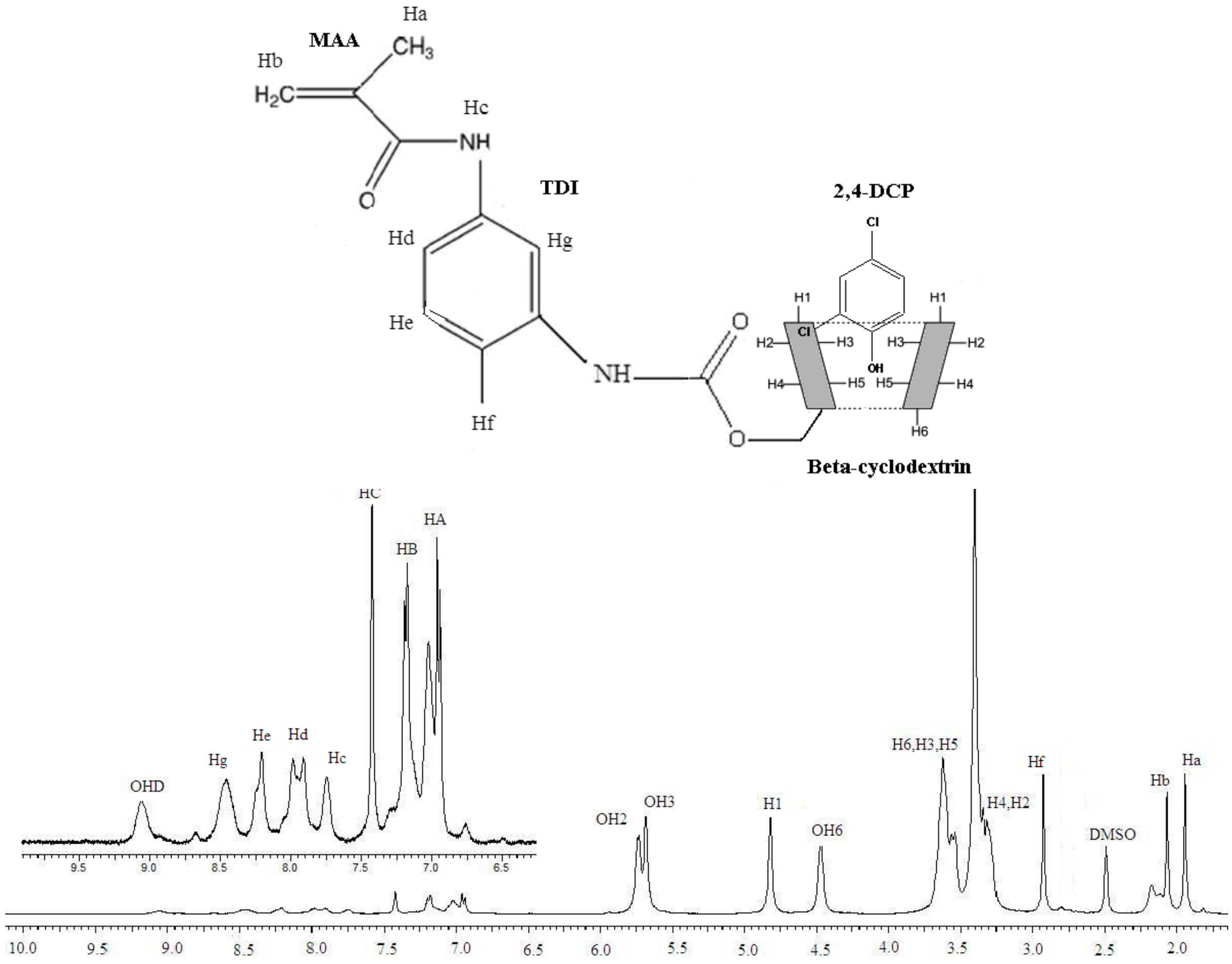

(**a**)

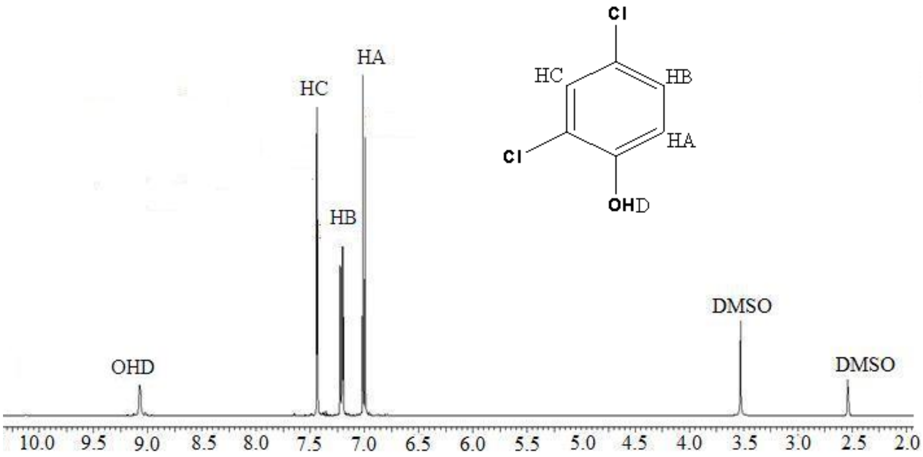

(**b**)

Figure S1. *Cont.*

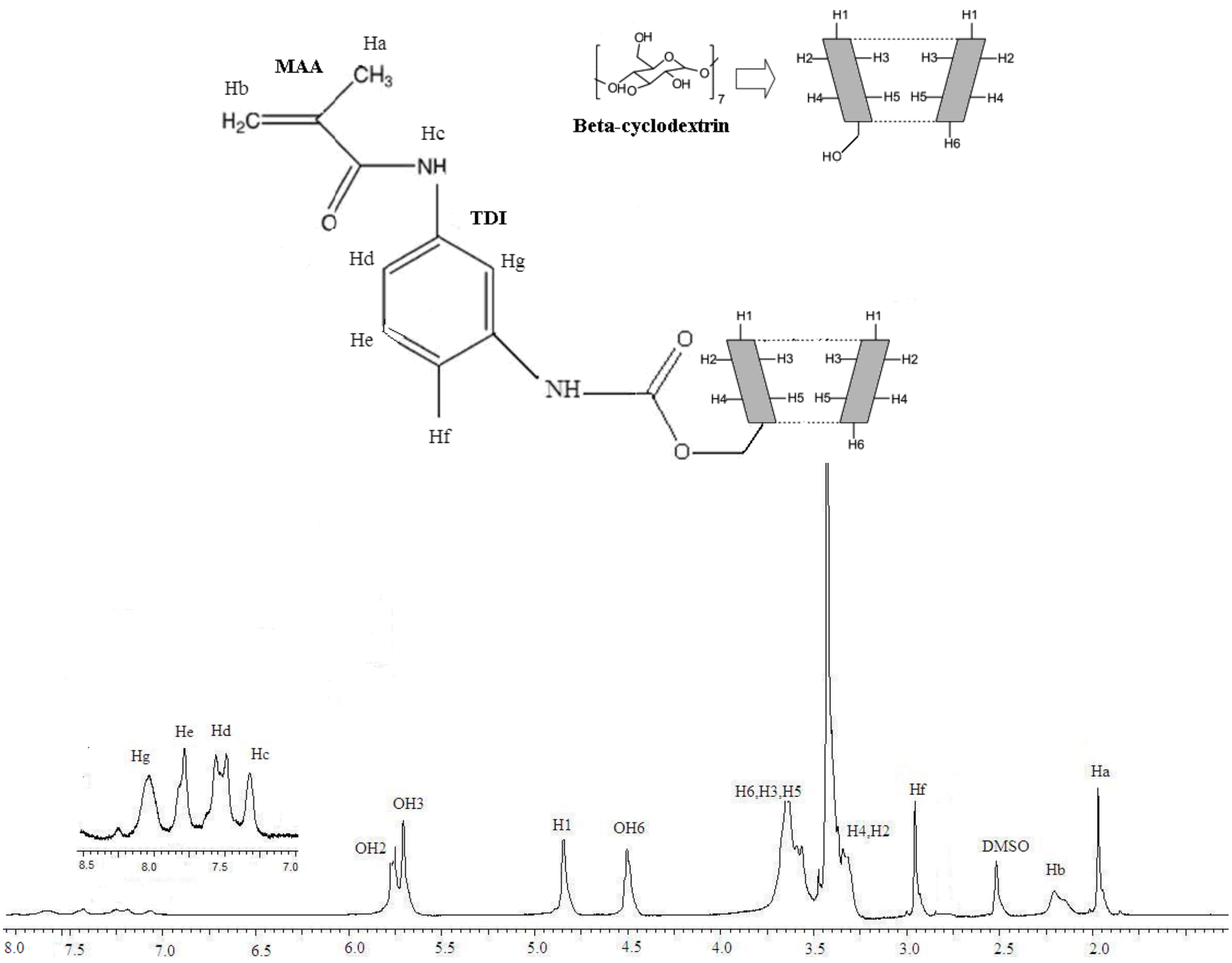

(c)

Table S1. ^{1}H NMR chemical shift (ppm) for MAA-βCD, 2,4-DCP and inclusion complex (MAA-βCD-DCP).

Compounds	MAA-βCD (δppm)	DCP (δppm)	MAA-βCD-DCP (δppm)	Δ MAA-βCD-DCP (δppm)
MAA				
Ha	1.9445		1.9408	−0.0037
Hb	2.7713		2.7676	−0.0037
TDI				
Hc	7.7248		7.7481	0.0233
Hd	7.9205		7.9079	−0.0126
He	8.2204		8.2088	−0.0116
Hf	2.9306		2.9245	−0.0061
Hg	8.4229		8.4547	0.0318

Table S1. *Cont.*

Compounds	MAA-βCD (δppm)	DCP (δppm)	MAA-βCD-DCP (δppm)	Δ MAA-βCD-DCP (δppm)
β-CD				
H_1	4.8203		4.8179	−0.0024
H_2	3.3272		3.3156	−0.0116
H_3	3.6213		3.5621	−0.0592
H_4	3.362		3.3406	−0.0214
H_5	3.6012		3.5395	−0.0617
H_6	3.6482		3.6231	−0.0251
OH_2	5.5806		5.7265	0.1459
OH_3	5.6752		5.6795	0.0043
OH_6	4.4487		4.4707	0.022
DCP				
H_A		6.9499	6.9377	−0.0122
H_B		7.1879	7.1952	0.0073
H_C		7.3697	7.4216	0.0519
OH_D		9.83	9.063	−0.767

Figure S2. Plot of Absorbance *versus* number of washing of template molecule (2,4-dichlorophenol).

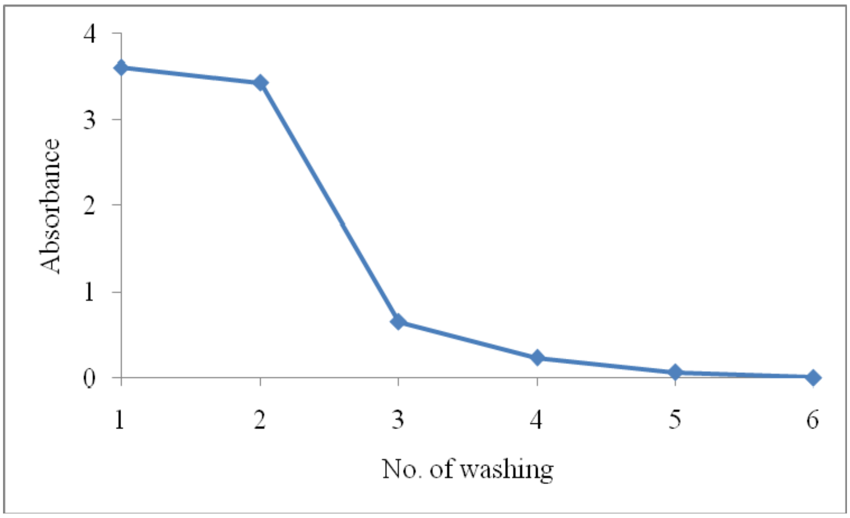

© 2014 by the authors; licensee MDPI, Basel, Switzerland. This article is an open access article distributed under the terms and conditions of the Creative Commons Attribution license (http://creativecommons.org/licenses/by/3.0/).
